# Death of Seurat

**DOI:** 10.3201/eid1101.AD1101

**Published:** 2005-01

**Authors:** Setu K. Vora

**Affiliations:** * New York Weill Cornell Medical Center, New York, New York, USA

**Keywords:** Francisco José de Goya y Lucientes, Lazarillo de Tormes, Death of Seurat, Setu Vora Georges Pierre Seurat

"A sudden stupid sickness carried him off in a few hours when he was about to triumph: I curse providence and death."

Art critic Jules Christophe, writing after the death of Seurat in La Plume, September 1, 1891

Born in Paris on December 2, 1859, Georges Pierre Seurat was only 31 when he died on March 29, 1891. In his short but productive life, this renowned painter founded a new art movement, neoimpressionism or pointillism. He is most famous for his work A Sunday on La Grande Jatte (1884), now at The Art Institute of Chicago. Art historian Richard Thompson puts Seurat's success in perspective: the 1880s were recognized by contemporaries as a decade of great excitement and innovation and are regarded today as one of the most salient periods of esthetic change. To have forged a new visual language in such challenging circumstances, while only in one's late 20s, was a remarkable achievement (*1*).

Seurat's early demise and the 1990s reemergence in Russia and neighboring countries of diphtheria, the infectious disease that probably killed him, invite closer examination of the circumstances that surround his death. The diseases affecting many of Seurat's contemporaries have been well documented, including the neuropsychiatric illness of van Gogh (*2*), visual impairment of Edgar Degas (*3*), dwarfism of Toulouse-Lautrec (*4*), and rheumatoid arthritis of Renoir (*5*). The circumstances and cause of Seurat's untimely death are not clearly understood and deserve medical scrutiny.

## Life and Times

Seurat was his parent's belated third child—a younger brother died at age 5. At age 16, he attended a municipal school of design run by the sculptor Justin Lequien, and in 1878, he joined the École des Beaux-Arts under Henri Lehmann, a student of the famous neoclassical painter Ingres. At age 20, Seurat served for a year with the 119th infantry regimen at Brest. His military recruitment papers describe him as follows: "Brown hair, brown eyes, average forehead, prominent nose, average mouth, round chin, oval face, 5 feet 10½ inch, no distinguishing mark" (*6*).

His friends, who included fellow artists Paul Signac, Charles Angrand, and Albert Dubois-Pillet, always described him as robust, "a solid being, a grenadier" (*7*). He smoked cigarettes, but it is not known if he drank alcohol or absinthe in excess. He attended periodic compulsory military training, as mentioned in telegraphic correspondence to Signac in August 1887: "…weather mild. Nobody in the streets in the evening (Province). Slight spleen. From 22 August to 18 September, 28 days…" (*1*). Slight spleen probably refers to his depressed mood or annoyance at the upcoming military training for 28 days rather than to any splenic problem.

Seurat made summer trips to coastal towns (Le Crotoy, Honfleur, Gravelines) (*6*). In 1889, he traveled to Belgium, where he exhibited at the Salon des Vingt in Brussels. After returning from this trip, he met Madeleine Knobloch, a 20-year-old model, and started secretly living with her (*6*). When Madeleine became pregnant with his child, they moved from his studio at 128 bis Boulevard de Clichy to a tiny room measuring 5 meters square in a quiet courtyard off the Passage de l'Elysee-des-Beaux-Arts. Seurat acknowledged the paternity of his son born on February 16, 1890, and entered the name of the child in the civil registers as Pierre Georges. At his exhibition in the Salon des Indépendants the same year, he showed his only known portrait of Madeleine Knobloch: Young Woman Powdering Herself. Yet, his family and close friends were still completely unaware of his mistress and child. According to one biographer, Seurat inherited secretive tendencies and preference for isolation from his father (*1*). He became distressed at the news of van Gogh's suicide and the death of his friend and follower Dubois-Pillet (*8*) of smallpox that August.

Madeleine Knobloch was pregnant again at the beginning of 1891. Seurat was hard at work painting The Circus. Art critic and author Arsène Alexandre, who was seeing Seurat regularly at this time, described his comings and goings, the way he climbed up the ladder and down again, how he worked long into the night (*9*). In early March, Seurat helped arrange the Indépendants exhibition, inspecting the entries and hanging the paintings. As if he had a premonition of death, he displayed his unfinished painting The Circus. He hardly noticed an ordinary sore throat that followed a cold (*10*). On March 26, he suddenly fell ill with fever and weakness. On March 27, Good Friday morning, he moved to his mother's apartment in the boulevard Magenta, supported by a friend and accompanied by pregnant Madeleine and their 13-month-old son. His illness was diagnosed as infectious angina or quinsy, and he was confined to bed. After a short crisis marked by fever and delirium, Seurat "choked to death" on Easter Sunday, March 29, at 6 a.m (*9*). His son Pierre George died of a similar illness on April 13, and was buried alongside Seurat in Père-Lachaise cemetery (*7*). Seurat's father died on May 24, cause unknown. Three generations of the Seurat family died within a span of 2 months.

## Cause of Death

"Infectious angina" (*6**,**7*) is most widely blamed, but a form of meningitis (*1*) or pneumonia is also mentioned as the cause of Seurat's death (*11*). Given the more common current association of angina with the heart and not the throat, it is likely that an art historian may have misinterpreted infectious angina to mean heart infection (*12*).

Pneumonia, especially pneumococcal pneumonia, can begin as upper respiratory infection followed by fever, weakness, and difficulty breathing. Severe disease leading to sepsis can cause delirium and even meningitis followed by death. Conversely, bacterial meningitis caused by *Neisseria meningitidis* often begins as sore throat, followed by fever and delirium progressing to death. However, in Seurat's case, description of infectious angina and the terminal event of choking to death suggest infectious upper airway obstruction. A variety of infections can produce this syndrome.

In the absence of clinicopathologic data, it is impossible to know the exact infectious cause of Seurat's death. Peritonsillar abscess, also known as quinsy, is the most common deep infection of the head and neck in adults. Peritonsillar abscess is most common in persons 20 to 40 years of age (*13*); however, it is less likely to cause upper airway obstruction, unless it is bilateral. Acute bacterial epiglottitis, caused primarily by *Haemophilus influenzae* type b, usually affects children (ages 2 to 7 years) but also occurs in adults. Onset is usually explosive, with sore throat, fever, and dyspnea progressing rapidly to dysphagia, pooling of oral secretions, and drooling. Abrupt deterioration occurs within a few hours and, in the absence of adequate treatment, results in death (*14*). The clinical course of Seurat's illness was over a period of 1 week, and his son died 2 weeks later, which makes acute epiglottitis caused by *H. influenzae* type b a less likely diagnosis.

Eminent biographer Jean Sutter was the first to suggest diphtheria as the cause of Seurat's death (*6*). Known variously as deadly angina, gangrenous angina, angina suffocant, and malignant angina, diphtheria was epidemic in France in the 19th century (*15*). Humans are the only known reservoir for *Corynebacterium diphtheriae*. The primary modes of spread are airborne droplets and direct contact with respiratory secretions. Most respiratory tract diphtheria occurs in the colder months in temperate climates and is associated with crowded indoor living conditions (*16*). After an incubation period of 2 to 4 days, signs and symptoms of inflammation can develop at various sites within the respiratory tract. Although diphtheria mainly affects children, according to William Osler, in his textbook published a year after Seurat's death, adults are also frequently affected (*17*). Osler also noted that diphtheria epidemics vary in intensity. While in some epidemics infection is mild and rarely fatal, in others it is characterized by wide extension of the pseudomembrane and tends to attack the larynx.

Biographic evidence mounts on the side of pharyngeal-tonsillar diphtheria with toxemia resulting in prostration and stupor. But the immediate cause of Seurat's death was probably extension of the laryngeal membrane, causing acute airway obstruction and asphyxiation.

## Contributing Factors

Although clinical details are not available, the death of Seurat's son from a similar illness, within 2 weeks of his death, suggests household transmission. Seurat lived with his mistress and son in very confined quarters. Overcrowding is a known factor in *C. diphtheriae* transmission (*18*).

The case of Madeleine Knobloch, the only family member who did not succumb to diphtheria, remains enigmatic. We have no details about her life, health, or personal habits (e.g., alcohol abuse), except for the fact that her second child died at or soon after birth. Madeleine herself died of cirrhosis of the liver at age 35 (*7*). An asymptomatic carrier and "disperser" state for diphtheria has been described (*19*), and Madeleine might have played such a role in the cold month of March in the confines of their tiny room.

Seurat was working extremely hard just before he became ill with sore throat. On an earlier occasion, art critic and collector Gustave Kahn saw him in the Boulevard de Clichy studio completing a big canvas "with zeal so strong and in a heat so oppressive that he lost pounds before it was finished" (*9*). Signac recounted that Seurat would often have only a snack for lunch, a croissant and a bar of chocolate, so that he would not waste valuable time (*1*). The combination of acute severe exertion, poor nutrition, and grueling work could increase susceptibility for upper respiratory infection (*20*). Signac, the closest friend, follower, and champion of Seurat, was close to the truth when he sadly concluded, "Our poor friend killed himself by overwork."

## Historical Context

Throughout the 19th century, diphtheria was common and had even found its place in contemporary art. Goya's painting Lazarillo de Tormes (1819), also known as el Garrotillo (Figure), shows a child suffering from diphtheria and the man attending him. Diphtheria especially affected children (death rate 40%–50%) (*21*). Necrosis of the mucosa of the upper respiratory track resulted in the formation of a pseudomembrane, which in turn obstructed the airway, causing asphyxia and death. The technique of tracheotomy evolved during the 19th century, prompted by the need to control diphtheria epidemics (*15*). In 1807, death of his nephew's son from diphtheria prompted Napoleon Bonaparte to stimulate diphtheria research by offering a grand prize. It was in this context that French physician Pierre Bretonneau (1778–1862) studied the disease and coined the word *la diphtherite* (Greek for leather, describing the pharyngeal membrane). He also formed the clinical case definition and performed the first tracheotomy in 1825 (*15*). In 1885, New York physician Joseph O'Dwyer introduced tracheal intubation for the treatment of severe diphtheria (*22*).

**Figure:**
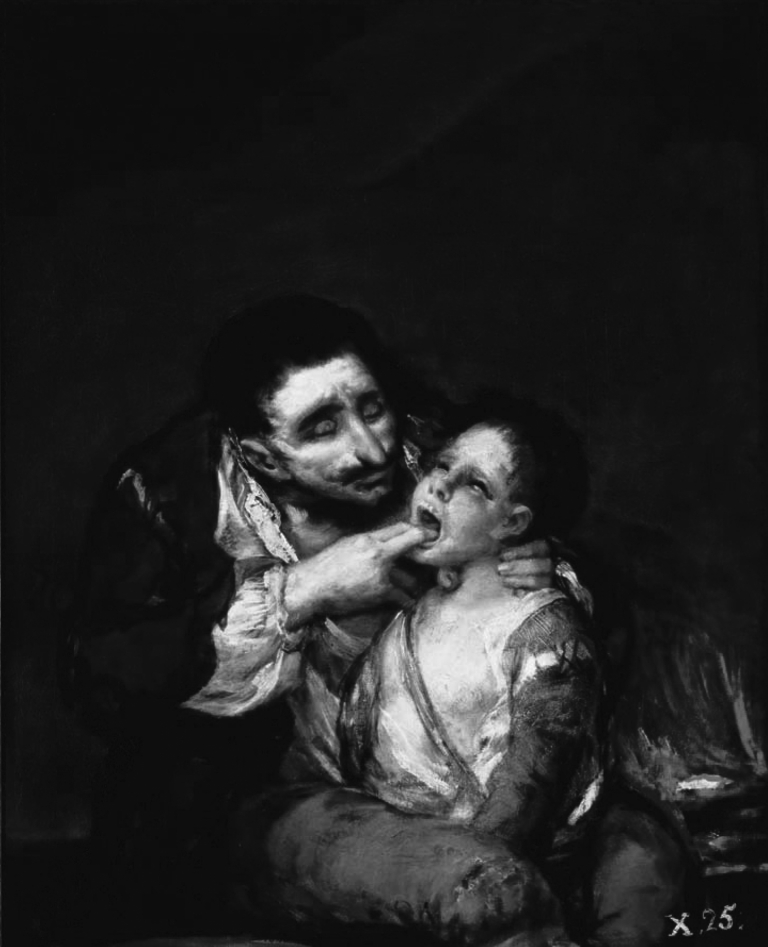
Francisco José de Goya y Lucientes (1746–1828). Lazarillo de Tormes (1819). Oil on canvas. Private Collection, Giraudon/Bridgeman Art Library/www.bridgeman.co.uk

Pierre Paul Emile Roux, an assistant of Louis Pasteur, proved that the diphtheria bacterium produced toxin (*23*). This discovery was instrumental to the development of antitoxin by Emil von Behring in 1890, for which Behring won the first ever Nobel Prize for physiology or medicine. On December 25, 1891, just a few months after Seurat's death, a patient with diphtheria was successfully treated with "immune serum" (*24*). Not much later, diphtheria was to touch and shape the life of another great artist. Picasso's 8-year-old sister died of the disease. While she was sick, Picasso vowed that if she survived, he would give up art. The relief he felt upon her death for not having to keep his promise left him with lifelong guilt. As his sister lay on her deathbed, she like the "sleeper" in many of his paintings and he like the "watcher," the child prayed to God, begging that she be spared. The event would govern his behavior and paintings for the rest of his life (*25*).

In the wake of the French Revolution, France had a national system of medical licensing in place by 1803 and the first true national healthcare system (*26*). Paris was the center of the medical world of France. Hotel Dieu, rebuilt during 1868–1878, and Salpetriere Hospital were among the leading hospitals providing healthcare to Parisians. The Pasteur Institute, established by decree on June 4, 1887, was inaugurated on November 14, 1888. France was leading research and treatment of diphtheria. Joseph Kinyoun, the founder of the Hygienic Laboratory—predecessor of the National Institutes of Health—learned the procedure for preparing diphtheria antitoxin at the Pasteur Institute in Paris. In France, tracheotomy had become the standard management for airway obstruction caused by diphtheria.

## Loss to the World

Seurat was not a struggling or impoverished artist who could not afford medical care. At a time when the average industrial worker was paid 150 francs a month, Seurat received a monthly allowance of 400 francs. He wore expensive top hats and black suits, which led Edgar Degas to dub him "le Notaire" (the Notary) (*6*). In spite of comfortable means and access to medically advanced Paris, Seurat chose to go to his mother's house and die there instead of going to a hospital, where tracheotomy or tracheal intubation might have saved him from asphyxiation. No record is available of Seurat's medical care during his lethal illness, and no autopsy was performed. We will never know what Seurat's achievements might have been if he had received medical treatment and lived to ripe old age, nor will we know if his son could have been a great artist himself.
